# Long-term follow-up study on nutritional problems and health-related quality of life among head and neck cancer survivors more than 5 years after diagnosis

**DOI:** 10.1007/s00520-026-10529-w

**Published:** 2026-03-11

**Authors:** Camilla Wallmander, Hedda Haugen Cange, Ewa Silander, Helen Larsson, Malin Börjesson, Leif Johansson, Ingvar Bosaeus, Eva Hammerlid

**Affiliations:** 1 Department of Otorhinolaryngology-Head and Neck Surgery, Institute of Clinical Sciences, Sahlgrenska Academy, University of Gothenburg, Gothenburg, Sweden; 2 Department of Otorhinolaryngology-Head and Neck Surgery, Sahlgrenska University Hospital, Region Västra Götaland, Gothenburg, Sweden; 3 Department of Oncology, Institute of Clinical Sciences, Sahlgrenska Academy, University of Gothenburg, Gothenburg, Sweden; 4 Department of Oncology, Sahlgrenska University Hospital, Region Västra Götaland, Gothenburg, Sweden; 5 Department of Otorhinolaryngology-Head and Neck Surgery, NU-Hospital Group, Region Västra Götaland, Trollhättan, Sweden; 6 Department of Otorhinolaryngology-Head and Neck Surgery, Södra Älvsborg Hospital Group, Region Västra Götaland, Borås, Sweden; 7 Department of Otorhinolaryngology-Head and Neck Surgery, Skaraborgs Hospital Group, Region Västra Götaland, Skövde, Sweden; 8 Department of Internal Medicine and Clinical Nutrition, Institute of Medicine, Sahlgrenska Academy, University of Gothenburg, Gothenburg, Sweden; 9 Department of Clinical Nutrition, Sahlgrenska University Hospital, Region Västra Götaland, Gothenburg, Sweden

**Keywords:** Head and neck cancer, Long-term survivors, Nutrition impact symptoms, Dietary adjustments, Muscle mass, Health-related quality of life

## Abstract

**Purpose:**

Few studies have evaluated the long-term effects of head and neck cancer (HNC) and its treatment. Therefore, the objective was to study nutritional rehabilitation needs by assessing nutritional problems, dietary adjustments, muscle mass, muscle strength, physical performance, prevalence of sarcopenia, and health-related quality of life (HRQoL) among long-term HNC survivors.

**Methods:**

This cross-sectional study included HNC survivors more than 5 years after diagnosis. Nutritional status, sarcopenia, and physical performance were assessed through questions about dietary adjustments, muscle mass (bioelectrical impedance analysis), grip strength, and maximum walking speed measurements. HRQoL and nutrition impact symptoms (NISs) were assessed using quality of life questionnaires from the European Organization for Research and Treatment of Cancer (EORTC), QLQ-C30 and QLQ-HN35, and were compared with reference values from a normal Swedish population.

**Results:**

Almost 80% of 114 participating survivors needed dietary adjustments, most commonly extra liquid with meals and/or moist food and increased time to consume meals. Relatively few patients had reduced muscle mass and low BMI, and none had sarcopenia. Compared with reference values, survivors reported severe HNC-specific symptoms on the EORTC QLQ-HN35. Survivors with the most problems swallowing solid food had a higher NIS burden and more problems with role and social functioning.

**Conclusions:**

In this cross-sectional study, many long-term HNC survivors experienced chronic NISs and had worse HRQoL than a matched reference group from the normal population. The findings suggest that survivors with nutritional problems may have adapted and used dietary adjustments to facilitate food intake. For some survivors, nutritional rehabilitation may be needed long after treatment has ended.

## Introduction

Head and neck cancer (HNC) includes tumors of the lip, oral cavity, salivary glands, oropharynx, hypopharynx, larynx, nasopharynx, and nasal sinuses. HNC is the seventh most common cancer diagnosis worldwide, with a 5-year survival rate of approximately 50–60%, which varies greatly depending on the tumor site and presents discrepancies across countries [1–3]. Treatment includes surgery, radiation, and chemotherapy, either alone or in combination [4]. Treatment side effects often lead to the development of nutrition impact symptoms (NISs), which affect the ability to eat, and common NISs are pain, dry mouth, sticky saliva, dysphagia, and problems with teeth, chewing, or opening the mouth wide [5, 6]. Nutritional problems can lead to the need for dietary adjustments, insufficient food intake, weight loss, loss of muscle mass, and malnutrition and can negatively affect health-related quality of life (HRQoL) [5–8]. It is common for patients to experience acute NISs before and during treatment, but complications can also persist after treatment and become chronic [5]. In HNC survivors, NISs and impaired HRQoL have been reported to persist for up to 5 years after diagnosis, with the greatest deterioration occurring during treatment [9–11]. There have been few long-term follow-up studies on nutritional problems, nutritional status, and HRQoL more than 5 years after diagnosis, and the majority have included a small sample of HNC survivors [12–18]. With the increasing incidence of HNC and improved survival rates, research focusing on the growing population of HNC survivors is warranted. The need for nutritional rehabilitation, e.g., the need for optimization of energy and nutrient intake, management of NISs and unfavorable changes in body weight, body composition, and function, after routine clinical tumor controls at specialist clinics have ceased has not been sufficiently researched [19]. The primary objective of this study was therefore to evaluate whether nutritional rehabilitation is needed for long-term HNC survivors diagnosed more than 5 years earlier, by assessing the need for dietary adjustments, use of oral nutritional supplements, and enteral nutrition, and to evaluate muscle mass, muscle strength, physical performance, sarcopenia, and HRQoL. The secondary aims were to describe and analyze a subgroup of participants with self-reported problems swallowing solid food and to compare the survivors’ HRQoL with reference values from a normal Swedish population.

## Method and materials

### Study design and study population

This cross-sectional long-term follow-up study of HNC survivors was conducted in four hospitals in western Sweden. This study is part of a multinational study conducted by the Quality of Life Group and the HNC Group of the European Organization for Research and Treatment of Cancer (EORTC), which aimed to describe late toxicity and HRQoL in HNC survivors, after clinical tumor controls have ceased at the specialist clinic 5 years after diagnosis [20]. The inclusion criteria were a diagnosis of cancer of the lip, oral cavity, oropharynx, nasopharynx, hypopharynx, larynx, salivary glands, or HNC of unknown primary (HNCUP) > 5 years earlier; ≥ 18 years of age; and the ability to attend clinics and complete questionnaires in Swedish. Patients with ongoing HNC or other cancers were excluded. Participants diagnosed from 2013 to 2015 were identified consecutively via the register available from the multidisciplinary tumor board at Sahlgrenska University Hospital. Information about the study was sent by mail to eligible participants, and after 1–2 weeks, they were contacted by telephone and asked about their participation. An invitation for a physical visit was sent by mail to those who agreed to participate. The participants received oral and written information and gave their written consent to participate in the study that took place between April 2021 and December 2021. The study visit included a clinical examination by a physician from the Department of Otorhinolaryngology or Oncology; measurements of body composition, muscle strength and physical performance; questions about dietary adjustments, oral nutritional supplements (ONSs), and enteral nutrition; and quality of life (QoL) questionnaires. The QoL questionnaires were sent to the participants in advance and returned at the study visit.

### Nutritional status and physical performance

Nutritional status and physical performance were assessed by measuring body weight, body composition, muscle strength, and walking speed. Body weight was measured to the nearest 0.1 kg, height was measured to the nearest 0.5 cm, and body mass index (BMI) was calculated. BMI was categorized according to cutoffs for underweight (< 18.5 kg/m^2^), normal weight (18.5–24.9 kg/m^2^), overweight (25–29.9 kg/m^2^), and obesity (> 30 kg/m^2^). A BMI < 20 kg/m^2^ for those aged < 70 years or < 22 kg/m^2^ for those aged ≥ 70 years is also often used as a cutoff for low BMI and was thus also presented [21]. Body composition was measured via bioelectrical impedance analysis (BIA) (Nutribox, Data Input, Lindenberg 7 82343 Pöcking, Germany) [22, 23]. BIA assessments were performed in a standardized manner with participants in the supine position, with two surface electrodes on the right hand and two on the right foot [23]. Participants were instructed not to eat or drink 2 h prior to the study visit, and before measurement was conducted, participants were required to lie supine for 5–10 min. The equation by Dey et al. [24] was used to calculate fat-free mass (FFM). The fat-free mass index (FFMI) was calculated by dividing the FFM (kg) by the height^2^ (m), and FFMI < 17 kg/m^2^ for men and < 15 kg/m^2^ for women were considered reduced muscle mass [21]. Muscle strength was assessed by measuring grip strength with a digital hand dynamometer (JAMAR® Plus+). The participants sat upright in a chair; in total, three attempts for each hand were performed, and the best result was recorded [25]. Hand grip strength < 27 kg for men and < 16 kg for women indicated reduced muscle strength [26]. Physical performance was assessed through a 10-m walk at maximum pace with acceleration and deceleration phases of 2 m each [27]. Participants were instructed to walk from the starting point to a visual cone placed at 14 m. Two attempts were made, and the best result was recorded. Sarcopenia was assessed using the diagnostic criteria of the European Working Group on Sarcopenia in Older People (EWGSOP2), where low muscle strength together with low muscle mass is required for diagnosis [26]. The study dietitian asked participants about the use of dietary adjustments to facilitate food intake, if they had an enteral feeding tube, and if they used oral nutritional supplements (ONSs). There were four questions about dietary adjustments: “Do you feel that it takes a long time to eat a meal?”; “Do you need to drink extra liquid with meals and/or do you need food to be moist?”; “Do you need to take small bites/chew carefully?”; “Do you need to adjust the texture of your food?” All the questions were based on participants’ perceptions of their need for dietary adjustments, and there was no definition of “a long time” in the question “Do you feel that it takes a long time to eat a meal?” All questions were dichotomous (yes or no), and if participants answered yes to a question, they were identified as having a need for that adjustment. Responses were not ranked if survivors answered yes to multiple questions.

### Health-related quality of life

Patients answered two HRQoL questionnaires from the *European Organization for Research and Treatment of Cancer* (EORTC) [28, 29]. The EORTC core questionnaire (EORTC QLQ-C30) consists of five functional scales, nine symptom scales, and one global QoL scale. The HNC module (EORTC QLQ-HN35) includes questions related to symptoms and problems specific to patients with HNC and includes 18 symptom scales. Both questionnaires have been validated and are used extensively in research. Most of the questions are answered on a 4-point Likert scale with the answers “not at all,” “a little,” “quite a bit,” and “very much.” Through linear transformation, the answers were converted to a score ranging from 0 to 100. High scores on the functional scales and on the global QoL scale indicated a high level of QoL, whereas high scores on the symptom scales indicated poor QoL with a high level of symptoms or problems. A difference in score of 10 or more points was considered to indicate a clinically relevant difference [30].

For this study, scales related to nutritional problems and general HRQoL were analyzed. From the EORTC QLQ-C30 scales regarding global QoL, physical, role, emotional, and social functioning, fatigue, and appetite loss were presented together with scales regarding problems with local pain, swallowing, senses, social eating, teeth, opening mouth, dry mouth, and sticky saliva from the EORTC QLQ-HN35. The participants’ HRQoL was compared to age- and sex-matched reference values from a normal Swedish population [31]. A subgroup representing participants with most difficulty swallowing solid food was analyzed separately and included participants who answered, “quite a bit” or “very much” to the question “Have you had problems swallowing solid food?” from the EORTC QLQ-HN35.

### Statistical analysis

Continuous variables are presented as the mean and standard deviation (SD). Categorical variables are presented as numbers and percentages (%). The reference values from the normal population were age- and sex-matched with those of the study group using a greedy group matching approach. Matching was performed by iteratively selecting the closest individuals from the normal population with the minimum *t* statistic tested against the study population. For the HRQoL questionnaires, at least 50% of the items on a scale needed to be answered to qualify for analysis. For comparisons between groups, Fisher’s exact test was used for dichotomous variables, the Mantel–Haenszel chi square test for ordered categorical variables, and the chi square test for nonordered categorical variables. Continuous variables were compared using Fisher’s nonparametric permutation test and the mean and SD are presented together with the mean difference and confidence interval. The threshold for statistical significance was a two-sided *p* value of 0.05. Analyses were performed via SAS*®* version 9.4 TS Level 1M6 (Cary, NC, USA).

## Results

### Patient characteristics

**Fig. 1:**
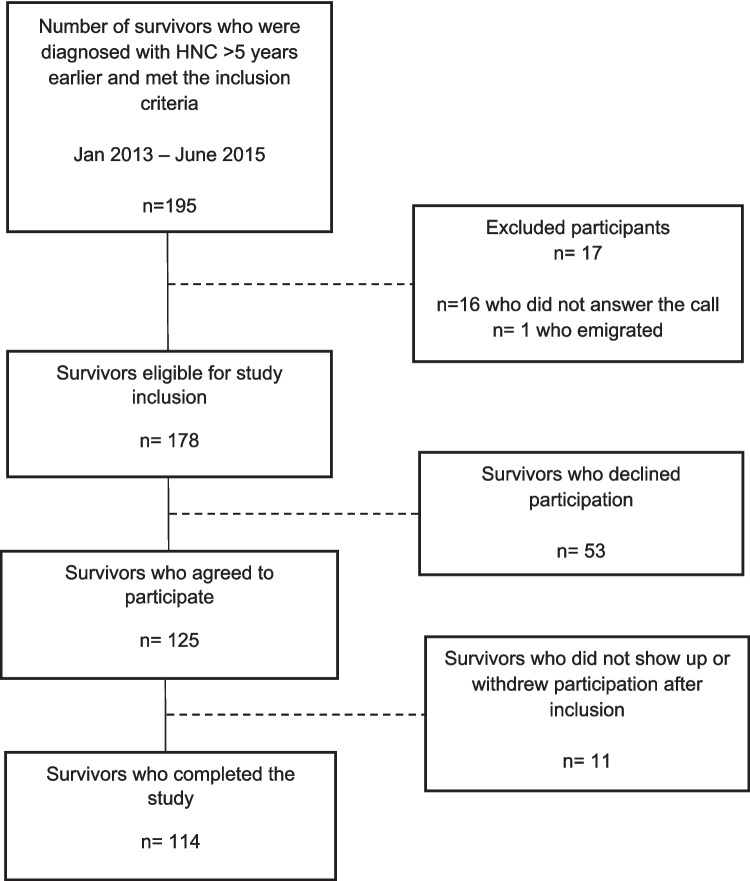
Flowchart of study inclusion

Among the 178 eligible survivors, 114 completed the study, and a flowchart of the inclusion process is presented in Fig. 1. Time since diagnosis ranged from 6.0 to 11.6 years. The mean age at the time of the study was 67 years and ranged from 38 to 89 years, and the majority were male (Table 1). The most common tumor sites were the oropharynx (46%) and oral cavity (29%). Compared with the entire study group, participants with oropharyngeal cancer were younger, and most of those in the oral cancer group were women. Stage III–IV cancer was most common in the study group, but stage I–II disease was most common among oral cancer survivors. Most participants had received radiotherapy (RT) + systemic therapy, followed by surgery alone and surgery + RT. Compared with the entire study group, more participants with oropharyngeal cancer received RT + systemic therapy, whereas most of the participants with oral cancer had been treated with surgery. The majority of participants had a Karnofsky performance status (KPS) score of 100, and most of the participants who lived alone and were former smokers had been diagnosed with oral cancer (Table 1).

**Table 1:** Participant characteristics

Variable	Total *n* = 114	Oropharynx *n* = 52	Oral cavity *n* = 33
Years since diagnosis	7.5 (SD 0.7)	7.6 (SD 0.5)	7.3 (SD 1.0)
Age (years at follow-up)	67.2 (SD 9.9)	65.1 (SD 7.6)	68.8 (SD 11.9)
< 70 years	65 (57)	39 (75)	12 (36)
≥ 70 years	49 (43)	13 (25)	21 (64)
Female	41 (36)	11 (21)	18 (54.5)
Male	73 (64)	41 (79)	15 (45.5)
Tumor site			
Oropharynx	52 (46)		
Oral cavity	33 (29)		
Larynx	10 (9)		
HNC of unknown primary	6 (5)		
Salivary glands	5 (4)		
Nasal cavity/sinus	4 (3)		
Hypopharynx	2 (2)		
Nasopharynx	2 (2)		
Stage*			
I–II	43 (38)	4 (8)	26 (79)
III–IV	71 (62)	48 (92)	7 (21)
Treatment**			
Surgery	23 (20)	0 (0)	21 (64)
Radiotherapy (RT)	12 (10)	5 (9)	0 (0)
Surgery + RT ± systemic therapy	27 (24)	3 (6)	12 (36)
RT + systemic therapy	52 (46)	44 (85)	0 (0)
Karnofsky performance status score			
100	68 (60)	33 (63.5)	20 (61)
90	27 (24)	12 (23)	6 (18)
80	14 (12)	6 (11.5)	4 (12)
70	4 (3)	1 (2)	2 (6)
60	1 (1)	0 (0)	1 (3)
Living situation			
Alone	39 (34)	14 (27)	16 (48)
Together with partner and/or children	75 (66)	38 (73)	17 (52)
Smoking			
Never smoker	51 (45)	29 (56)	11 (33)
Former smoker	59 (52)	22 (42)	19 (58)
Current smoker	4 (3)	1 (2)	3 (9)

For continuous variables, the mean (SD) is presented, and for categorical variables, the number (%) is presented.

*SD* standard deviation

*Staging according to the Union for International Cancer Control TNM classification of malignant tumors, 7th edition

**Treatment: 10 participants had received treatment for recurrence. Out of the 50 participants receiving surgery, 36 had resection of the primary tumor and neck dissection, 10 had only primary tumor resection, and 4 only neck dissection. In total, 15 reconstructions (regional or free flap) were performed; 11 as part of the primary treatment, and 4 as part of recurrence treatment. Out of 91 participants receiving radiation, 87 were treated with IMRT and 4 participants with larynx cancer received 3D conformal RT. Out of 62 patients receiving systemic therapy, 55 were treated with cisplatin, 5 with cetuximab, and 2 with cisplatin and cetuximab. Two patients were treated with laryngectomy—one hypopharyngeal cancer and one oropharyngeal cancer after recurrence. For the remaining two participants with oropharyngeal cancer who received surgery, one received neck dissection, and one underwent resection of recurrence, including reconstruction with free flap and neck dissection

### Nutritional status and physical performance

**Fig. 2:**
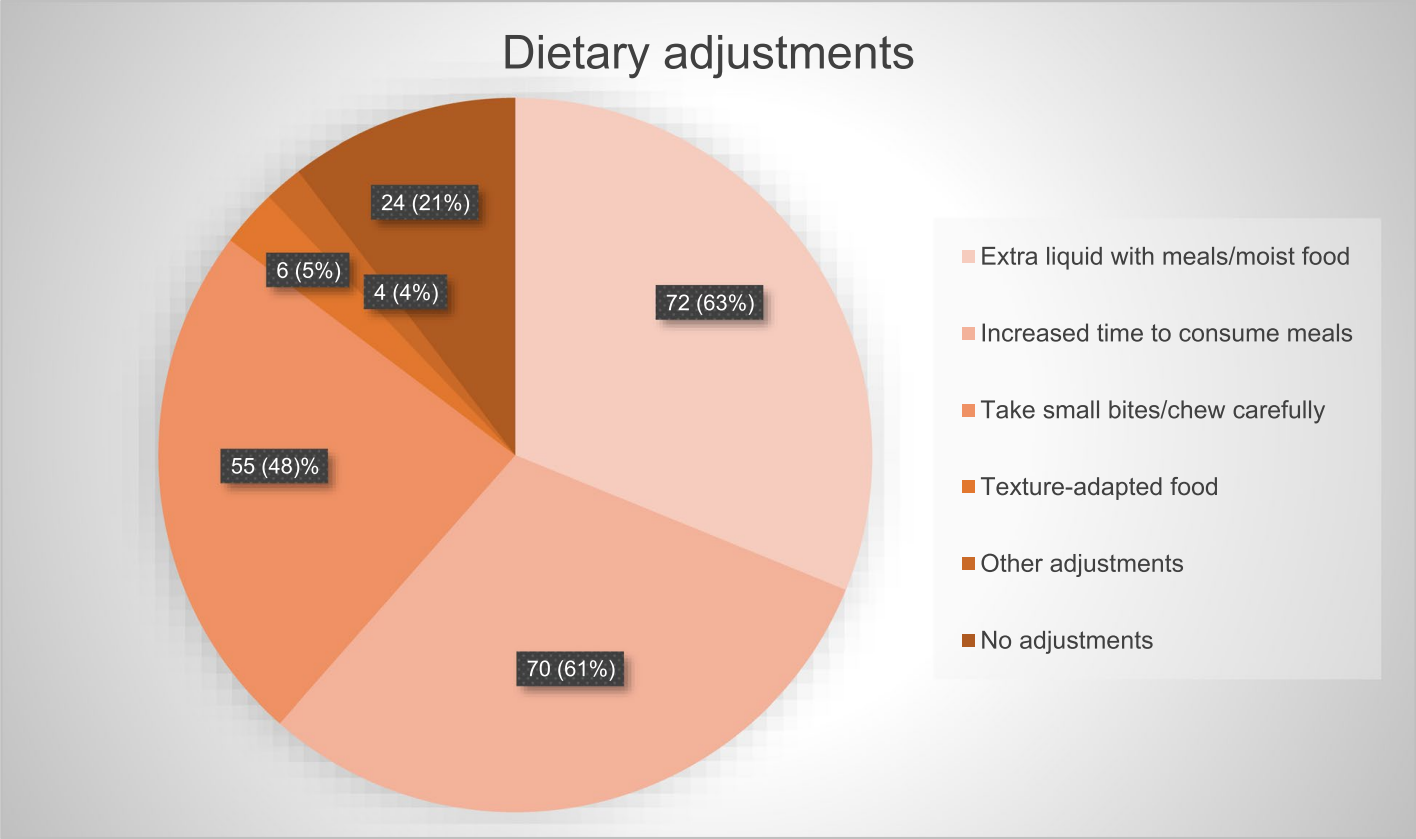
Among 114 participants, 87 reported a need for dietary adjustments to facilitate food intake. These 87 survivors reported a total of 207 adjustments. Three dietary adjustments were most common, followed by 2 adjustments. The answers to the questions about adjustments were solely based on the participants’ perceptions. “Other adjustments” included the use of saliva gel and not eating warm food. The three patients with PEG could not answer the questions, as their oral intake was nonexistent or sparse (taste portions), and they were not included in the figure. Two of these three participants stated that the small amount of food taken orally needed to be adjusted to a puree or liquid consistency

In total, 87 participants reported that they used dietary adjustments to facilitate food intake (Fig. 2). Thirty-five participants reported using three adjustments to facilitate food intake, while 30 participants reported a need for two adjustments. The two most common adjustments were the need for extra liquid with meals and/or the need for moist food and increased time to consume meals. Only six participants reported that they needed to adjust the texture of the food; of those, five needed mashed/soft food, and one needed pureed food (Fig. 2). Twelve participants used ONSs regularly as a complement to their diet, three participants received nutrition via percutaneous endoscopic gastrostomy (PEG), and 16 participants had contact with a dietitian at least yearly.

The participants’ body weight ranged widely from 45.5 to 141 kg, their BMI ranged from 17.9 to 48.1 kg/m^2^, and approximately 50% were considered overweight or obese (Table 2). The FFMI ranged from 13.9 to 23.7 kg/m^2^, and seven women and seven men were considered to have reduced muscle mass. In total, 20 participants had a low BMI and/or reduced muscle mass. Of those 20, seven had contact with a dietitian. Muscle strength varied from 13.1 to 67.2 kg, and one female and one male were considered to have reduced muscle strength. Maximum walking speed ranged from 0.8 to 4.1 m/s. No participants were diagnosed with sarcopenia. More participants with lower average weight, low BMI, and reduced FFMI were observed in the oral cancer group. The average muscle mass, muscle strength, and maximum walking speed were similar between the two tumor groups (Table 2).

**Table 2:** Nutritional status and physical performance for the total study population and for the tumor locations of the oropharynx and oral cavity

	Total *n *= 114	Oropharynx *n* = 52	Oral cavity *n* = 33
Body weight (kg)	76.2 (16.4)	78.0 (12.8)	73.0 (22.2)
BMI (kg/m^2^)	25.3 (4.0)	25.2 (3.1)	24.8 (5.1)
BMI categories, *n* (%)			
< 18.5	2 (2%)	0 (0%)	2 (6%)
18.5–24.9	57 (50%)	27 (52%)	19 (58%)
25–29.9	41 (36%)	22 (42%)	7 (21%)
> 30	14 (12%)	3 (6%)	5 (15%)
Low BMI, *n* (%)	16 (14%)	4 (8%)	9 (27%)
FFMI (kg/m^2^)			
Male	19.2 (1.8)	19.1 (1.4)	19.4 (3.0)
Female	16.4 (1.4)	16.4 (1.1)	16.2 (1.1)
Reduced FFMI, *n* (%)	14 (12%)	3 (6%)	7 (21%)
Muscle strength (max value, kg)			
Male	45.1 (9.5)	46.0 (7.9)	44.4 (13.5)
Female	26.7 (6.4)	28.5 (8.0)	26.3 (6.1)
10-m max walk (m/s)			
Male	2.2 (0.6)	2.6 (0.5)	2.1 (0.5)
Female	1.9 (0.5)	2.1 (0.5)	1.9 (0.4)

For continuous variables, the mean (SD) is presented, and for categorical variables, the number (%) is presented. Low BMI =  < 20 kg/m^2^ for age < 70 years and < 22 kg/m^2^ for age ≥ 70 years. Reduced FFMI =  < 17 kg/m^2^ for men and < 15 kg/m^2^ for women. Missing values: FFMI *n* = 5.

*n* number, *BMI* body mass index, *FFMI* fat-free mass index

### Health-related quality of life

Compared with the age- and sex-matched reference values from a normal population, the study participants reported both clinically and significantly greater symptom burden on all scales from the EORTC QLQ-HN35 (Table 3). From the EORTC QLQ-C30, only appetite loss differed significantly, with more problems in the study group than in the reference group, but the difference did not reach 10 points. Prominent problems in the entire study group and in the specific tumor location groups were dry mouth and sticky saliva, with the highest scores reported by the survivors in the oropharyngeal cancer group. Compared with the survivors of oropharyngeal cancer, the participants with oral cancer reported more appetite loss, with a 7.9-point difference. Moreover, problems with swallowing were more severe in the oropharyngeal cancer group, whereas problems with teeth were worse in the oral cancer group (Table 3).

**Table 3:** Health-related quality of life for the total study population and for the tumor locations of the oropharynx and oral cavity, including comparisons to reference values from a normal population

	Total *n* = 113	Reference values *n* = 456	*p* value	Mean difference and CI	Oropharynx *n* = 51	Oral cavity *n* = 33
**EORTC QLQ-C30**						
Global QoL	74.1 (21.0)	76.3 (21.8)	0.35	−2.2 (−6.5; 2.4)	77.6 (22.3)	73.0 (19.1)
Physical functioning	88.6 (14.7)	86.7 (20.2)	0.34	1.9 (−1.9; 6.0)	92.8 (11.7)	84.2 (17.5)
Role functioning	88.6 (22.9)	84.8 (27.0)	0.17	3.8 (−1.5; 9.4)	92.0 (19.1)	86.4 (25.2)
Emotional functioning	84.1 (20.9)	85.1 (19.8)	0.60	−1.1 (−5.1; 3.2)	86.4 (20.3)	82.8 (20.2)
Social functioning	87.0 (23.8)	89.1 (21.5)	0.39	−2.0 (−6.4; 2.6)	87.3 (22.8)	88.9 (22.3)
Fatigue	20.6 (22.9)	20.0 (22.0)	0.76	0.7 (−4.0; 5.1)	18.1 (22.0)	22.2 (22.9)
Appetite loss	8.3 (18.7)	4.1 (13.9)	*0.0091*	4.2 (1.1; 7.1)	5.2 (12.2)	13.1 (24.9)
**EORTC QLQ-HN35**						
Local pain	13.4 (18.4)	2.9 (8.9)	< *0.0001*	10.5 (8.0; 12.7)	13.4 (17.3)	16.5 (21.7)
Swallowing	18.6 (19.5)	2.3 (7.9)	< *0.0001*	16.3 (13.9; 18.5)	23.5 (18.2)	13.1 (19.5)
Senses	17.3 (25.5)	5.5 (15.4)	< *0.0001*	11.7 (7.9; 15.2)	17.3 (26.0)	15.7 (21.2)
Social eating	14.1 (21.3)	2.9 (10.2)	< *0.0001*	11.2 (8.3; 13.7)	12.1 (19.0)	17.0 (23.6)
Teeth	21.4 (31.6)	10.4 (21.7)	*0.0002*	11.1 (5.9; 15.9)	17.0 (30.1)	27.3 (32.8)
Opening mouth	19.0 (27.5)	1.9 (11.1)	< *0.0001*	17.2 (13.8; 20.3)	19.6 (26.0)	19.2 (31.2)
Dry mouth	48.5 (35.2)	14.4 (24.2)	< *0.0001*	34.1 (28.5; 39.5)	54.9 (32.5)	44.4 (36.0)
Sticky saliva	37.8 (33.0)	7.6 (18.5)	< *0.0001*	30.2 (25.5: 34.8)	43.1 (33.5)	26.3 (29.8)

For continuous variables, the mean (SD) is presented. For comparisons between the entire study group and the age- and sex-matched reference values from a Swedish normal population, the Fisher’s nonparametric permutation test for continuous variables was used and presented with the mean difference and CI. EORTC QLQ-C30 and QLQ-HN35: A higher score for the global quality of life scale and the function scales reflects better function, and a higher score for the symptom scales represents more problems. Differences between the two groups were considered statistically significant if *p *< 0.05, and clinically relevant if >10 points.

*CI* confidence interval, *EORTC QLQ-C30* The European Organization for Research and Treatment of Cancer Quality of Life Questionnaire-Core 30, *EORTC QLQ-HN35* The European Organization for Research and Treatment of Cancer Quality of Life Questionnaire-Head and Neck 35

### Comparison between survivors with or without problems swallowing solid food

The participants who reported “quite a bit” or “very much” on the question “Have you had problems swallowing solid food?” from the EORTC QLQ-HN35 (*n* = 26) were compared to the remaining study group (*n* = 86); two participants did not answer the question (Table 4). Among the 86 participants, 46 reported no problems, and 40 answered “a little” to the question “Have you had problems swallowing solid food?”. Lower KPS scores were more prevalent among the participants with swallowing difficulties. Age, sex, tumor site, stage, and treatment did not differ significantly, but the group with swallowing problems tended to have more survivors with stage III–IV oropharyngeal cancer and multimodal treatment (Table 4).

**Table 4:** Comparison of demographics between survivors with or without problems swallowing solid food

	Problems swallowing solid food *n* = 26	No problems swallowing solid food *n* = 86	*p* value
Age at study follow-up (years)	66.7 (SD 11.1)	67.4 (SD 9.7)	0.77
< 70 years	15 (58)	49 (57)	
≥ 70 years	11 (42)	37 (43)	1.00
Female	8 (31)	33 (38)	
Male	18 (69)	53 (62)	0.64
Tumor site			
Oropharynx	14 (54)	37 (43)	
Oral cavity	6 (23)	27 (31)	
Larynx	2 (7.5)	7 (8)	
HNC of unknown primary	2 (7.5)	4 (5)	
Salivary glands	1 (4)	4 (5)	
Nasal cavity/sinus	1 (4)	3 (4)	
Hypopharynx	0 (0)	2 (2)	
Nasopharynx	0 (0)	2 (2)	0.92
Stage*			
I–II	8 (31)	37 (43)	
III–IV	18 (69)	49 (57)	0.11
Treatment**			
Surgery	1 (4)	22 (26)	
Radiotherapy (RT)	4 (15)	7 (8)	
Surgery + RT ± systemic therapy	7 (27)	21 (24)	
RT + systemic therapy	14 (54)	36 (42)	0.096
Karnofsky performance status			
100	13 (50)	55 (64)	
90	7 (27)	18 (21)	
80	2 (7.5)	12 (14)	
70	3 (11.5)	1 (1)	
60	1 (4)	0 (0)	*0.048*

Participants with swallowing problems defined by reporting “quite a bit” or “very much” on the question “Have you had problems swallowing solid food?” from the EORTC QLQ-HN35. For continuous variables, the mean (SD) is presented, and for categorical variables, the number (%) is presented. For comparisons between the groups, Fisher’s exact test was used for dichotomous variables, the Mantel–Haenszel chi-square test for ordered categorical variables, chi-square test for nonordered categorical variables, and Fisher’s nonparametric permutation test for continuous variables. Differences between the two groups were considered statistically significant if *p* <0.05.

*SD* standard deviation

*Staging according to the Union for International Cancer Control TNM classification of malignant tumors, 7th edition

All participants with most difficulties swallowing solid food reported a need for dietary adjustments to facilitate food intake, and the need for three adjustments (61%) was most common. In comparison, 73% of the participants with no or few swallowing difficulties needed to adapt their food intake, with the need for two adjustments (29%) being the most common. In both groups, the most common adjustments were the need for extra liquid with meals and/or the need for moist food and increased time to consume meals. In the group with swallowing problems, seven survivors had contact with a dietitian (including the three survivors with PEG) and four participants used ONSs. None of the nutritional or physical performance variables differed significantly between the two groups (Table 5). However, a greater proportion of participants in the group with swallowing problems had a low BMI and a reduced FFMI (Table 5).

**Table 5:** Comparisons of nutritional status, physical performance, and health-related quality of life between survivors with or without problems swallowing solid food

	Problems swallowing solid food *n* = 26	No problems swallowing solid food *n* = 86	*p* value	Mean difference and CI
BMI categories (kg/m^2^),* n* (%)				
< 18.5	1 (4%)	1 (1%)	0.84	
18.5–24.9	13 (50%)	44 (51%)		
25–29.9	9 (34.5%)	32 (37%)		
> 30	3 (11.5%)	9 (11%)		
Low BMI, *n* (%)	6 (23%)	10 (12%)	0.26	
FFMI (kg/m^2^)				
Male	18.9 (1.6)	19.3 (1.9)	0.47	−0.4 (−1.4; 0.6)
Female	16.4 (2.0)	16.4 (1.2)	0.93	−0.05 (−1.2; 1.1)
Reduced FFMI, *n* (%)	5 (19%)	9 (11%)	0.45	
Muscle strength (max value, kg)				
Male	45.0 (10.4)	45.3 (9.1)	0.92	−0.3 (−5.5; 4.8)
Female	24.6 (4.4)	27.3 (6.7)	0.29	−2.7 (−7.9; 2.2)
10-m max walk (m/s)				
Male	2.1 (0.4)	2.3 (0.6)	0.29	−0.2 (−0.5; 0.1)
Female	1.7 (0.5)	2.0 (0.5)	0.23	−0.2 (−0.6; 0.2)
**EORTC QLQ-C30**				
Global QoL	72.1 (23.7)	75.0 (20.1)	0.56	−2.9 (−12.0; 6.6)
Physical functioning	85.1 (19.4)	89.6 (13.0)	0.20	−4.5 (−10.8; 2.2)
Role functioning	79.5 (33.1)	91.4 (18.1)	*0.037*	*−11.9 (−21.3; −1.0)*
Social functioning	75.6 (31.4)	90.3 (20.0)	*0.011*	*−14.7 (−24.6; −4.2)*
Fatigue	27.4 (29.0)	18.5 (20.5)	0.11	8.9 (−1.7; 18.5)
Appetite loss	12.8 (19.0)	7.0 (18.5)	0.24	5.8 (−3.2; 13.3)
**EORTC QLQ-HN35**				
Local pain	20.2 (18.6)	11.5 (18.0)	*0.048*	*8.7 (0.3; 16.3)*
Senses	25.6 (32.1)	14.7 (22.8)	0.073*	10.9 (−0.8; 21.7)
Social eating	31.7 (22.2)	8.91 (18.1)	< *0.0001*	*22.8 (13.9; 30.8)*
Teeth	33.3 (37.7)	17.8 (28.8)	*0.045*	*15.5 (1.6; 28.8)*
Opening mouth	35.9 (33.9)	14.0 (23.1)	*0.0010*	*21.9 (10.0; 33.3)*
Dry mouth	60.3 (32.7)	45.0 (35.3)	0.063*	15.3 (0.0; 31.2)
Sticky saliva	50.0 (38.0)	34.1 (30.7)	*0.040*	*15.9 (1.7; 30.2)*

Participants with swallowing problems defined by reporting “quite a bit” or “very much” on the question “Have you had problems swallowing solid food?” from the EORTC QLQ-HN35. For continuous variables, the mean (SD) is presented, and for categorical variables, the number (%) is presented. For comparisons between the groups, Fisher’s exact test was used for dichotomous variables and the Mantel–Haenszel chi-square test for ordered categorical variables. The Fisher’s nonparametric permutation test was used for continuous variables and is presented together with the mean difference and CI. EORTC QLQ-C30 and QLQ-HN35: A higher score for the global quality of life scale and the function scales reflects a better function, and a higher score for the symptom scales represents more problems. Differences in HRQoL variables between the two groups were considered clinically relevant if >10 points. Differences between the two groups were considered statistically significant if *p* <0.05.

*BMI* body mass index, *CI* confidence interval, *FFMI* fat-free mass index, *EORTC QLQ-C30* The European Organization for Research and Treatment of Cancer Quality of Life Questionnaire-Core 30, *EORTC QLQ-HN35* The European Organization for Research and Treatment of Cancer Quality of Life Questionnaire-Head and Neck 35

*No significant difference but a clinically relevant difference

The participants with swallowing problems reported significantly worse role and social functioning on the EORTC QLQ-C30 (Table 5). They also reported more problems with fatigue, but the difference of 9 points was not statistically significant. For the EORTC QLQ-HN35, the group with swallowing problems reported more problems on all seven scales, and the differences for five of the scales were also statistically significant. The greatest differences between the two groups were found for trouble with social eating and problems with opening the mouth (Table 5).

## Discussion

Survivors of HNC are a growing population facing many challenges, and the long-term effects of HNC and its treatment have not been sufficiently researched. To our knowledge, this is one of the first studies to evaluate and describe survivors’ nutritional problems, muscle mass, muscle strength, physical performance, and HRQoL more than 5 years after diagnosis. Survivors were assessed an average of 7.5 years after diagnosis. Almost 80% of participants used dietary adjustments, which is consistent with previous findings from qualitative studies, where HNC survivors, who were mainly assessed within 5 years post-treatment, reported a need for extra liquid, increased time to consume meals, smaller bites, and cutting food into smaller pieces [32–34]. Surprisingly, only six participants in our study reported the need to adjust the texture of the food, suggesting that the other adjustments were sufficient to facilitate eating. Almost 25% of the survivors reported difficulty swallowing solid food, and all of these participants required dietary adjustments. In a large study from the EORTC working group, difficulty swallowing was the second most common self-reported problem among long-term survivors of HNC [35], and in a systematic review of NISs in HNC survivors, dysphagia was the most common long-term side effect described after chemoradiotherapy [5]. These findings suggest that dysphagia and the need for dietary adjustments may persist for a long time and may indicate a need for nutritional support more than 5 years after diagnosis.

The HRQoL of the survivors was compared with age- and sex-matched reference values from a normal population, and the participants scored worse on all scales of the QLQ-HN35, which was consistent with the results observed in a previous study with a follow-up of 5 years after treatment [36]. The HRQoL reported by HNC survivors in the study by Abel et al. [36] was comparable to that of our participants, except for problems with loss of appetite, senses, and dry mouth, for which there was a tendency for fewer symptoms in our study. The high burden of nutrition impact symptoms observed on the QLQ-HN35 might also explain why many patients needed to adjust their food intake. Dry mouth and sticky saliva were pronounced problems in the study group, and a systematic review of nearly 1400 oropharyngeal cancer survivors found clinically important deterioration of these symptoms at least 1 year after treatment (range 1–26 years) [37]. For the two most common tumor locations in the study, oropharyngeal and oral cancer, the results of the QLQ-HN35 were either equal or better for the participants with oral cancer, except for problems with teeth. This difference may be explained by the treatment regimen; most of the participants diagnosed with oropharyngeal cancer had advanced cancer and received combination therapy, whereas the majority in the oral cancer group had stage I–II cancer and received treatment with a single modality. Similarly, the EORTC 1629 study that compared HRQoL between single and multimodal treatments for long-term HNC survivors revealed no differences in functional scale scores, but survivors treated with a single modality reported similar or lower symptom burden [20].

Relatively few participants in the study group used ONS and enteral nutrition, and relatively few had a low BMI, reduced muscle mass, and muscle strength, and none had sarcopenia. Furthermore, no differences in these variables were observed between participants with the most problems swallowing solid food and survivors without swallowing difficulties. Additionally, the mean values for grip strength and maximum walking speed were comparable to reference values [27, 38]. These findings may imply that many survivors may adapt and compensate for their NISs with adjustments to facilitate food intake, and we speculate that this adaptation allows the majority to meet energy and nutritional needs and thereby maintain body mass and function. The survivors with most problems swallowing solid food had a greater need for dietary adjustments, poorer social and role function, and significantly greater NIS burden. Subgroups of survivors with affected swallowing ability, high NIS burden, and reduced muscle mass may be nutritionally vulnerable, which may imply a need for nutritional rehabilitation. Adaptation to nutritional problems may also result in a “new normal,” where survivors do not recognize that they are still suffering from long-term side effects from treatment [34, 39]. Healthcare professionals may also have to ask questions about dietary adjustments to identify HNC survivors who might need nutritional treatment. In contrast to our results, a pilot study revealed that a lower NIS burden after RT was associated with greater muscle mass and better functional, physical, emotional, and total quality of life [40]. Nevertheless, comparison is difficult since the sample size of the pilot study was small, and the follow-ups varied from 6 months to 9 years after diagnosis. Although the findings in our study suggest that many survivors compensate for their nutritional problems, we do not know whether diet quality might be affected. A hypothesis for future research could be to investigate whether long-term nutritional problems experienced by HNC survivors may lead to negative effects on nutritional intake, for example, a high intake of saturated fat and low intake of vegetables, fruit, and fiber. Other studies have reported lower diet quality scores among HNC survivors than reference values and that higher diet quality is associated with a lower NIS burden and better survival [40–42].

The strengths of this long-term follow-up study are that all HNC survivors were evaluated more than 5 years after diagnosis, and the number of participants included was relatively large, which is uncommon in this patient population. To our knowledge, this was also one of the first long-term follow-ups to evaluate body composition, muscle strength, physical performance, and sarcopenia. Limitations include the cross-sectional study design, with no information about causality or changes over time. No information was collected on survivors who declined participation, and we do not know whether these differed from the study group, which poses a risk of selection bias. The questions about dietary adjustments were based on extensive clinical experience but were not validated. The subgroups that were based on tumor location and swallowing difficulties were relatively small, and the analyses were explorative, which may have affected the generalizability of the study.

## Conclusion

In conclusion, this cross-sectional study revealed that many long-term survivors of HNC, on average 7.5 years after diagnosis, experienced chronic nutrition impact symptoms and worse HRQoL than a matched reference group from the normal population. Relatively few patients used ONSs and enteral nutrition, and relatively few were considered to have reduced BMI, muscle mass, and muscle strength. The findings suggest that the majority of survivors with nutritional problems may have developed coping strategies and used dietary adjustments to ease food intake. However, for some survivors, nutritional rehabilitation may be needed long after treatment has ended, focusing on nutrition impact symptoms related to HRQoL and the management of eating difficulties.

## Data Availability

The data that support the findings of this study are available upon request from the corresponding author. The data is not publicly available because of privacy or ethical considerations.

## References

[1] Bray F, Laversanne M, Sung H, Ferlay J, Siegel RL, Soerjomataram I, Jemal A (2024) Global cancer statistics 2022: GLOBOCAN estimates of incidence and mortality worldwide for 36 cancers in 185 countries. CA Cancer J Clin 74:229–263. 10.3322/caac.21834 38572751 10.3322/caac.21834

[2] Giraldi L, Leoncini E, Pastorino R et al (2017) Alcohol and cigarette consumption predict mortality in patients with head and neck cancer: a pooled analysis within the International Head and Neck Cancer Epidemiology (INHANCE) Consortium. Ann Oncol 28:2843–2851. 10.1093/annonc/mdx486 28945835 10.1093/annonc/mdx486 PMC5834132

[3] Gatta G, Luttmann S, Trama A, Rossi S, Galceran J, Innos K, Guevara M, Licitra L, Bennet D, Redondo-Sánchez D, Capocaccia R (2025) Epithelial head and neck cancer survival in Europe: geographical variation, time trends and long term survival. Eur J Cancer 229:115692. 10.1016/j.ejca.2025.115692 40945302 10.1016/j.ejca.2025.115692

[4] Johnson DE, Burtness B, Leemans CR, Lui VWY, Bauman JE, Grandis JR (2020) Head and neck squamous cell carcinoma. Nat Rev Dis Primers 6:92. 10.1038/s41572-020-00224-3 33243986 10.1038/s41572-020-00224-3 PMC7944998

[5] Crowder SL, Douglas KG, Yanina Pepino M, Sarma KP, Arthur AE (2018) Nutrition impact symptoms and associated outcomes in post-chemoradiotherapy head and neck cancer survivors: a systematic review. J Cancer Surviv 12:479–494. 10.1007/s11764-018-0687-7 29556926 10.1007/s11764-018-0687-7

[6] Granström B, Holmlund T, Laurell G, Fransson P, Tiblom Ehrsson Y (2022) Addressing symptoms that affect patients’ eating according to the Head and Neck Patient Symptom Checklist(©). Support Care Cancer 30:6163–6173. 10.1007/s00520-022-07038-x 35426524 10.1007/s00520-022-07038-x PMC9135877

[7] Einarsson S, Karlsson HE, Björ O, Haylock AK, Tiblom Ehrsson Y (2020) Mapping impact factors leading to the GLIM diagnosis of malnutrition in patients with head and neck cancer. Clin Nutr ESPEN 40:149–155. 10.1016/j.clnesp.2020.09.174 33183529 10.1016/j.clnesp.2020.09.174

[8] Bressan V, Stevanin S, Bianchi M, Aleo G, Bagnasco A, Sasso L (2016) The effects of swallowing disorders, dysgeusia, oral mucositis and xerostomia on nutritional status, oral intake and weight loss in head and neck cancer patients: a systematic review. Cancer Treat Rev 45:105–119. 10.1016/j.ctrv.2016.03.006 27010487 10.1016/j.ctrv.2016.03.006

[9] Abendstein H, Nordgren M, Boysen M, Jannert M, Silander E, Ahlner-Elmqvist M, Hammerlid E, Bjordal K (2005) Quality of life and head and neck cancer: a 5 year prospective study. Laryngoscope 115:2183–2192. 10.1097/01.Mlg.0000181507.69620.14 16369164 10.1097/01.MLG.0000181507.69620.14

[10] Hammerlid E, Silander E, Hörnestam L, Sullivan M (2001) Health-related quality of life three years after diagnosis of head and neck cancer- -a longitudinal study. Head Neck 23:113–125. 10.1002/1097-0347(200102)23:2<113::aid-hed1006>3.0.co;2-w 11303628 10.1002/1097-0347(200102)23:2<113::aid-hed1006>3.0.co;2-w

[11] Aghajanzadeh S, Tuomi L, Karlsson T (2023) A 5-year prospective study of health-related quality of life in irradiated head and neck cancer patients: three trends of HRQL over time. Eur Arch Otorhinolaryngol 280:2617–2622. 10.1007/s00405-022-07789-7 36627402 10.1007/s00405-022-07789-7 PMC10066084

[12] Karsten RT, van der Molen L, Hamming-Vrieze O, van Son R, Hilgers FJM, van den Brekel MWM, Stuiver MM, Smeele LE (2020) Long-term swallowing, trismus, and speech outcomes after combined chemoradiotherapy and preventive rehabilitation for head and neck cancer; 10-year plus update. Head Neck 42:1907–1918. 10.1002/hed.26120 32112600 10.1002/hed.26120

[13] Kraaijenga SA, Oskam IM, van der Molen L, Hamming-Vrieze O, Hilgers FJ, van den Brekel MW (2015) Evaluation of long term (10-years+) dysphagia and trismus in patients treated with concurrent chemo-radiotherapy for advanced head and neck cancer. Oral Oncol 51:787–794. 10.1016/j.oraloncology.2015.05.003 26027851 10.1016/j.oraloncology.2015.05.003

[14] Yan YB, Meng L, Liu ZQ, Xu JB, Liu H, Shen J, Zhang XW, Peng X, Mao C (2017) Quality of life in long-term oral cancer survivors: an 8-year prospective study in China. Oral Surg Oral Med Oral Pathol Oral Radiol 123:67–75. 10.1016/j.oooo.2016.09.006 27876377 10.1016/j.oooo.2016.09.006

[15] Bjordal K, Kaasa S, Mastekaasa A (1994) Quality of life in patients treated for head and neck cancer: a follow-up study 7 to 11 years after radiotherapy. Int J Radiat Oncol Biol Phys 28:847–856. 10.1016/0360-3016(94)90104-x 8138437 10.1016/0360-3016(94)90104-x

[16] Mehanna HM, Morton RP (2006) Deterioration in quality-of-life of late (10-year) survivors of head and neck cancer. Clin Otolaryngol 31:204–211. 10.1111/j.1749-4486.2006.01188.x 16759240 10.1111/j.1749-4486.2006.01188.x

[17] Kraaijenga SA, van der Molen L, Jacobi I, Hamming-Vrieze O, Hilgers FJ, van den Brekel MW (2015) Prospective clinical study on long-term swallowing function and voice quality in advanced head and neck cancer patients treated with concurrent chemoradiotherapy and preventive swallowing exercises. Eur Arch Otorhinolaryngol 272:3521–3531. 10.1007/s00405-014-3379-6 25381096 10.1007/s00405-014-3379-6

[18] Ottosson S, Lindblom U, Wahlberg P, Nilsson P, Kjellén E, Zackrisson B, Levring Jäghagen E, Laurell G (2014) Weight loss and body mass index in relation to aspiration in patients treated for head and neck cancer: a long-term follow-up. Support Care Cancer 22:2361–2369. 10.1007/s00520-014-2211-6 24687537 10.1007/s00520-014-2211-6

[19] Goyal N, Day A, Epstein J et al (2022) Head and neck cancer survivorship consensus statement from the American Head and Neck Society. Laryngoscope Investig Otolaryngol 7:70–92. 10.1002/lio2.702 35155786 10.1002/lio2.702 PMC8823162

[20] Taylor KJ, Amdal CD, Bjordal K et al (2024) Long-term health-related quality of life in head and neck cancer survivors: a large multinational study. Int J Cancer 154:1772–1785. 10.1002/ijc.34861 38312044 10.1002/ijc.34861

[21] Cederholm T, Jensen GL, Correia M et al (2019) GLIM criteria for the diagnosis of malnutrition - a consensus report from the global clinical nutrition community. Clin Nutr 38:1–9. 10.1016/j.clnu.2018.08.002 30181091 10.1016/j.clnu.2018.08.002

[22] Kyle UG, Bosaeus I, De Lorenzo AD et al (2004) Bioelectrical impedance analysis - part I: review of principles and methods. Clin Nutr 23:1226–1243. 10.1016/j.clnu.2004.06.004 15380917 10.1016/j.clnu.2004.06.004

[23] Kyle UG, Bosaeus I, De Lorenzo AD et al (2004) Bioelectrical impedance analysis-part II: utilization in clinical practice. Clin Nutr 23:1430–1453. 10.1016/j.clnu.2004.09.012 15556267 10.1016/j.clnu.2004.09.012

[24] Dey DK, Bosaeus I, Lissner L, Steen B (2003) Body composition estimated by bioelectrical impedance in the Swedish elderly. Development of population-based prediction equation and reference values of fat-free mass and body fat for 70- and 75-y olds. Eur J Clin Nutr 57:909–916. 10.1038/sj.ejcn.1601625 12879085 10.1038/sj.ejcn.1601625

[25] Roberts HC, Denison HJ, Martin HJ, Patel HP, Syddall H, Cooper C, Sayer AA (2011) A review of the measurement of grip strength in clinical and epidemiological studies: towards a standardised approach. Age Ageing 40:423–429. 10.1093/ageing/afr051 21624928 10.1093/ageing/afr051

[26] Cruz-Jentoft AJ, Bahat G, Bauer J et al (2019) Sarcopenia: revised European consensus on definition and diagnosis. Age Ageing 48:16–31. 10.1093/ageing/afy169 30312372 10.1093/ageing/afy169 PMC6322506

[27] Bohannon RW (1997) Comfortable and maximum walking speed of adults aged 20–79 years: reference values and determinants. Age Ageing 26:15–19. 10.1093/ageing/26.1.15 9143432 10.1093/ageing/26.1.15

[28] Aaronson NK, Ahmedzai S, Bergman B, Bullinger M, Cull A, Duez NJ, Filiberti A, Flechtner H, Fleishman SB, de Haes JC et al (1993) The European Organization for Research and Treatment of Cancer QLQ-C30: a quality-of-life instrument for use in international clinical trials in oncology. J Natl Cancer Inst 85:365–376. 10.1093/jnci/85.5.365 8433390 10.1093/jnci/85.5.365

[29] Bjordal K, Hammerlid E, Ahlner-Elmqvist M, de Graeff A, Boysen M, Evensen JF, Biörklund A, de Leeuw JR, Fayers PM, Jannert M, Westin T, Kaasa S (1999) Quality of life in head and neck cancer patients: validation of the European Organization for Research and Treatment of Cancer Quality of Life Questionnaire-H&N35. J Clin Oncol 17:1008–1019. 10.1200/jco.1999.17.3.1008 10071296 10.1200/JCO.1999.17.3.1008

[30] Osoba D, Rodrigues G, Myles J, Zee B, Pater J (1998) Interpreting the significance of changes in health-related quality-of-life scores. J Clin Oncol 16:139–144. 10.1200/jco.1998.16.1.139 9440735 10.1200/JCO.1998.16.1.139

[31] Hammerlid E, Adnan A, Silander E (2017) Population-based reference values for the European Organization for Research and Treatment of Cancer Head and Neck module. Head Neck 39:2036–2047. 10.1002/hed.24870 28708279 10.1002/hed.24870

[32] Crowder SL, Najam N, Sarma KP, Fiese BH, Arthur AE (2020) Head and neck cancer survivors’ experiences with chronic nutrition impact symptom burden after radiation: a qualitative study. J Acad Nutr Diet 120:1643–1653. 10.1016/j.jand.2020.04.016 32646742 10.1016/j.jand.2020.04.016

[33] Einarsson S, Laurell G, TiblomEhrsson Y (2019) Experiences and coping strategies related to food and eating up to two years after the termination of treatment in patients with head and neck cancer. Eur J Cancer Care (Engl) 28:e12964. 10.1111/ecc.12964 30444049 10.1111/ecc.12964

[34] Ganzer H, Rothpletz-Puglia P, Byham-Gray L, Murphy BA, Touger-Decker R (2015) The eating experience in long-term survivors of head and neck cancer: a mixed-methods study. Support Care Cancer 23:3257–3268. 10.1007/s00520-015-2730-9 25851804 10.1007/s00520-015-2730-9

[35] Taylor KJ, Amdal CD, Bjordal K et al (2023) Serious long-term effects of head and neck cancer from the survivors’ point of view. Healthcare. 10.3390/healthcare11060906 36981562 10.3390/healthcare11060906 PMC10048748

[36] Abel E, Silander E, Nyman J, Björk-Eriksson T, Hammerlid E (2020) Long-term aspects of quality of life in head and neck cancer patients treated with intensity modulated radiation therapy: a 5-year longitudinal follow-up and comparison with a normal population cohort. Adv Radiat Oncol 5:101–110. 10.1016/j.adro.2019.07.015 32051896 10.1016/j.adro.2019.07.015 PMC7004944

[37] Høxbroe Michaelsen S, Grønhøj C, Høxbroe Michaelsen J, Friborg J, von Buchwald C (2017) Quality of life in survivors of oropharyngeal cancer: a systematic review and meta-analysis of 1366 patients. Eur J Cancer 78:91–102. 10.1016/j.ejca.2017.03.006 28431302 10.1016/j.ejca.2017.03.006

[38] Dodds RM, Syddall HE, Cooper R et al (2014) Grip strength across the life course: normative data from twelve British studies. PLoS One 9:e113637. 10.1371/journal.pone.0113637 25474696 10.1371/journal.pone.0113637 PMC4256164

[39] Ottosson S, Laurell G, Olsson C (2013) The experience of food, eating and meals following radiotherapy for head and neck cancer: a qualitative study. J Clin Nurs 22:1034–1043. 10.1111/jocn.12151 23480499 10.1111/jocn.12151

[40] Crowder SL, Li Z, Sarma KP, Arthur AE (2021) Chronic nutrition impact symptoms are associated with decreased functional status, quality of life, and diet quality in a pilot study of long-term post-radiation head and neck cancer survivors. Nutrients. 10.3390/nu13082886 34445046 10.3390/nu13082886 PMC8401587

[41] Maino Vieytes CA, Mondul AM, Crowder SL, Zarins KR, Edwards CG, Davis EC, Wolf GT, Rozek LS, Arthur AE (2021) Pretreatment adherence to a priori-defined dietary patterns is associated with decreased nutrition impact symptom burden in head and neck cancer survivors. Nutrients. 10.3390/nu13093149 34579024 10.3390/nu13093149 PMC8464702

[42] Maino Vieytes CA, Rodriguez-Zas SL, Madak-Erdogan Z, Smith RL, Zarins KR, Wolf GT, Rozek LS, Mondul AM, Arthur AE (2022) Adherence to a priori-defined diet quality indices throughout the early disease course is associated with survival in head and neck cancer survivors: an application involving marginal structural models. Front Nutr 9:791141. 10.3389/fnut.2022.791141 35548563 10.3389/fnut.2022.791141 PMC9083460

